# Research and recovery: Can patient participation in research promote recovery for people with complex post‐traumatic stress disorder, CPTSD**?**

**DOI:** 10.1111/hex.13014

**Published:** 2019-12-23

**Authors:** Catherine Matheson, Elizabeth Weightman

**Affiliations:** ^1^ Senior Psychotherapist South London and Maudsley NHS Mental Health Trust London UK; ^2^ Department of Psychology University of Exeter Exeter UK

**Keywords:** complex post‐traumatic stress disorder, CPTSD, liberation psychology, participatory research, refugees, sexual abuse

## Abstract

**Background:**

A new diagnosis of complex post‐traumatic stress disorder, CPTSD, has been agreed by the World Health Organization, WHO, and evidence is needed for what psychological treatment might be effective, particularly from those with experience of the disorder. We used a novel participatory approach to explore patient views and simultaneously studied the impact on the patient researchers of the research process itself. In this paper, we report on the latter section of the study how the involvement in research of patients with CPTSD affected their mental health. Symptoms of CPTSD may include emotional dysregulation, feelings of self‐worthlessness and difficulties in relationships.

**Objective:**

The aim of this study section was to explore whether patients’ mental health could be promoted through empowering them to participate in research on CPTSD.

**Design:**

The study had a qualitative, participatory design. The clinician who led the research (first author) held group meetings with patient researchers to explore the impact of the research process. The clinician also kept notes on the process in a reflective log.

**Setting and participants:**

Six patient researchers participated in research with other patients with lived experience of CPTSD in an NHS outpatient unit in a London hospital.

**Intervention studied:**

The research process itself was analysed in group meetings with researchers which the clinician recorded and transcribed.

**Findings:**

Participation in research may promote increased self‐confidence and social inclusion for those with CPTSD.

**Conclusion:**

Involvement in research may be seen as an empowering intervention because patients felt it contributed to recovery.

## 1 INTRODUCTION

When we chose a participatory approach to studying psychological treatment for the new diagnosis of complex post‐traumatic stress disorder, CPTSD, we also wanted to explore whether research participation itself might help to overcome the symptoms described in the ICD‐11^1^ as ‘particular difficulties in sustaining relationships’, as well as ‘persistent beliefs about oneself as diminished, defeated or worthless’. This was because a wide range of existing research on the mental health advantages of research participation has shown that it can promote social interaction and personal development.^2^, ^3^, ^4^

The relevant literature on participatory research could be categorized into two broad fields, with different, but overlapping, perspectives. The first is the community psychology field where participation in research is seen as a catalyst to promote social learning and social change.^5^, ^6^ The second is the service user research field which focuses on more democratic and better quality knowledge production.^7^, ^8^, ^9^, ^10^, ^11^ They share a critical attitude towards intrapsychic explanations for poor mental health and look instead towards power inequalities in society.

Community psychology has ideological roots in international community development where the first author (initials) previously worked. Early writers such as the Brazilian educator, Paolo Freire,^12^ and the Salvadoran social psychologist, Ignacio Martín‐Baró,^6^, ^13^ were interested in promoting popular participation in research as a means to empower marginalized social sectors. Martín‐Baró, the founder of liberation psychology, wrote specifically about the impact of violence and trauma on mental health in the 1980s. For instance, he did a quantitative study involving participants in opinion polls during the civil war in El Salvador, which allowed people to express views too dangerous to voice publicly. It is necessary to note here that Martín‐Baró challenged the medical model of psychiatric diagnosis and felt that symptoms could sometimes be more accurately seen as ‘a normal reaction to an abnormal situation’.^13^

More recently, Kagan et al^5^ have applied liberation psychology concepts to social marginalization in the UK and how psychologists might promote mental health through encouraging participatory research and community action. Their 2002 paper^5^ mentioned studies involving single parents, young people with Tourette syndrome, socially excluded older men and traveller communities in Spain, but only one brief summary concerned mental health service users who implemented an evaluation of community mental health services.

The second field of relevant literature emerged in the UK in the 1990s when a growing service user movement in mental health focused specifically on empowerment for service users as a marginalized sector.^4^, ^10^ The service user movement argued that health professionals’ epistemological approach to evidence, with its emphasis on randomized controlled trials, RCTs, had become too dominant at the expense of service users’ knowledge from experience. They pointed out that service user input into research could result in more effective treatment that would correspond more closely with patients’ needs and preferences.^11^ In research terms, it may also lead to fuller and more honest information if patients feel more able to disclose their views to other patients.^7^

Frost et al^7^ bring together the two theoretical strands outlined above and describe combining the philosophies of community‐based participatory research with patient and public involvement, PPI, into ‘emancipatory practices’. This includes taking an approach where those who are the focus of the research take on the researcher role. The intervention studied was to facilitate patient agenda setting in clinical consultations on diabetes. King and Gillard^14^ also applied ideas from development studies in their work where academics and community co‐researchers collaborated in an evaluation of a primary care mental health service. They discuss how this meant ‘a re‐alignment of power within research relationships’^14^ (p. 2) and a recognized need to integrate local knowledge and experience into the research process.

The most relevant study in the literature was by Springham and colleagues,^15^ who organized a research network of service users in south London. It was intended to promote recovery through social inclusion, as well as learning or recovering work skills. The study included reflections by service user researchers on the benefits to their mental health of taking part in the research network. They had a range of diagnoses, and symptoms of CPTSD were not specified.

The brief summary of relevant literature above suggests that participatory research may lead to mental health improvements for different groups of marginalized people, through facilitating personal development and social interaction. They included some who had experienced trauma.

The clinical literature on complex post‐traumatic stress disorder describes difficulties which might also be addressed by such transformative change.^16^ The diagnosis includes symptoms of core PTSD, which are re‐experiencing traumatic events, avoidance and hypervigilance.^17^ In addition, CPTSD is characterized by problems in emotional regulation; feelings of self‐worthlessness; and difficulties in sustaining relationships. People who have experienced prolonged trauma due to human mistreatment, such as torture, domestic violence or childhood abuse, may develop such difficulties.

Many clinicians advocate a three‐phase approach for psychological treatment which was originally formulated by Judith Herman in 1992,^18^ although this has recently been contested.^19^, ^20^ Herman described an initial stabilization phase, a trauma‐focused stage and a community re‐integration stage. From this perspective, participating in research could be seen as community integration during which relationships with others are rebuilt.^21^ This was seen in our study through opportunities for researchers to build relationships in group training, group supervision and data analysis groups, as well as individual interviews with other patients. There is an interesting overlap here with the more political perspective of social psychologists, such as Kagan et al,^5^ who advocate collective action.

Such empowerment may be particularly relevant for people with a CPTSD diagnosis given the symptom of believing oneself to be ‘diminished, defeated or worthless’.^17^

The objective of this study was to explore whether and how patient participation in research may promote recovery from CPTSD. It constituted a study of the participatory approach used in a larger study to explore patient views on psychotherapy for CPTSD, and these results are reported elsewhere.^22^

## 2 METHODS

### 2.1 Design

The literature on participatory research in health divides methods into three categories: user‐led research, collaboration and consultation.^9^, ^15^ This study comes under the category of collaboration between service users (experts by experience) and professionals (experts by learning). It is one section of a larger project in which former patients were recruited and trained by a psychotherapist to participate in design, data collection and analysis, to explore patient views of psychotherapy for CPTSD. In this paper, we take a step back to focus on this participatory approach itself, using similar collaborative methods. The methods were that while researchers were implementing interviews, the clinician held regular group meetings with them, including an analysis meeting at the end to explore how they felt about the research process. All the meetings were transcribed by the clinician, and the most significant themes were identified by the researchers. This data set is the focus of this paper. In addition, the clinician‐researcher kept a reflective log from a personal perspective in the interests of transparency, and the log was also considered as data.

### 2.2 Setting

The study was organized by a psychotherapist (first author) as part of her doctoral research. She leads the trauma service within an NHS secondary mental health team at an outpatient unit in (University Hospital Lewisham) in London. Referrals come from local community mental health teams and primary care teams in the Improving Access to Psychological Therapies, IAPT, service. The NICE‐recommended treatments for core PTSD are offered: trauma‐focused cognitive behavioural therapy, CBT, and eye movement desensitization and reprocessing, EMDR. A range of other modalities may also be provided including a stabilization group for Sri Lankan refugees held in a community garden and a therapy group for women with childhood experiences of sexual abuse.

The borough of Lewisham has an ethnically diverse population with 46% of people from a Black, Asian or Minority Ethnic background, BAME,^23^ with high levels of poverty, according to the Lewisham Poverty Commission Report.^24^ From 2013 to 2017, for the trauma service alone, an average of 75 referrals a year with a possible CPTSD diagnosis was received from primary care and community mental health teams. Black, Asian and Minority Ethnic referrals were the majority, reflecting significant numbers of refugees and asylum seekers in the borough.

### 2.3 Participants

The selection criteria for researchers were as follows:

- Not currently in employment.
- Sufficient English to take part in researcher training.
- More than 12 weeks since end of therapy.
- Completed at least 12 sessions of therapy.
- Currently resident in Lewisham.

All had a diagnosis of complex PTSD at assessment as defined by ICD‐11. This was measured by scores on Impact of Events Scale‐Revised, IES‐R,^25^ which is a self‐report questionnaire used as a screening measure for core PTSD. Narrative assessments were then searched to identify the additional three symptom clusters for complex PTSD described in ICD‐11: difficulties in regulating emotions; beliefs about oneself as diminished, defeated or worthless; and difficulties in sustaining relationships.

The reason for inviting those not in employment was in order to offer a training opportunity to people who were not working. An ability to communicate in English was also necessary for researcher selection because it was not possible to provide a translator for the training sessions. (This did not prevent participation by those who did not have English as a first language, who included three of the six researchers.)

The reason for waiting 12 weeks after the end of treatment is that some evidence suggests patients may be vulnerable soon after ending treatment.^26^ In order to have as realistic a view as possible of patient profile, patients with co‐morbid disorders including dissociation, substance misuse or personality disorder were included.

### 2.4 Researcher recruitment

Twenty‐seven invitation letters and information sheets were sent out to possible researchers who fulfilled the criteria, and follow‐up e‐mails and phone calls were made to discuss the research and answer questions. They were selected in chronological order from a total of 235 referrals to the trauma service over 3 years between October 2014 and September 2017. Eleven of those invited were from Sri Lanka, reflecting the high concentration of Sri Lankan Tamil refugees among patients referred, but none wanted to take on the researcher role. Some were working, but others did not give a reason. After the researchers were recruited, they decided that they would first interview each other to ensure that they had their own input into the research as former patients. There were 6 patient researchers who did 24 interviews.

### 2.5 Ethical considerations

There was a range of ethical concerns, and a number of writers were consulted about ethical standards in patient and public involvement, PPI, including Pandya‐Wood et al^27^ and Troya et al^28^ These considerations are detailed below.

#### 2.5.1 Psychotherapist researching own patients

The ethics of implementing a research project involving former patients included the possibility of systematic bias on the part of the psychotherapist, the conflicts and coincidences in the roles of researcher and psychotherapist, and the unequal power dynamic between former patients and psychotherapist as researcher and professional. These concerns were addressed in the design and methods of the study, including the participatory approach, the researchers’ reflections on their experience and the clinician's reflective log. Ethical approval was granted by NHS Westminster Research Ethics Committee reference 17/LO/1391.

#### 2.5.2 Ethics of patients implementing research

Other ethical concerns included the care and payment of former patients doing the research, as well as those being interviewed. Training was provided for service user researchers in ethical conduct and procedures such as safeguarding, confidentiality, personal limits and financial probity. They also had to complete the necessary police checks for working with vulnerable people through the Disclosure and Barring Service, DBS. These concerns were addressed by including researchers in the existing volunteer scheme run by the South London and Maudsley NHS Mental Health Trust, SLaM. Being a volunteer also meant that research activities could count as work experience for which a reference could be provided, thus helping participants into employment if they wished. Some of the researchers had not worked for some years.

#### 2.5.3 Patient after‐care

As former service users, the researchers may have been vulnerable to emotional disturbance when discussing treatment with participants. Regular post‐interview group meetings were organized for them at which such issues could be discussed. These meetings had a dual purpose in that they were also themselves studied to explore how researchers felt about the research process.

#### 2.5.4 Access for non‐English speakers

It was considered an ethical issue that the research did not exclude the views of non‐English speakers. The majority of patients seeking treatment for complex PTSD in Lewisham have English as a second language, although most speak adequate English for psychotherapy treatment. Researchers were provided with a translator for 5 participants of whom 4 were Tamil refugees from Sri Lanka.

#### 2.5.5 Payment for researchers

There may be ethical concerns about the financial exploitation of service user researchers, and a budget was secured to pay them £10 an hour for doing the interviews and attending meetings, if they chose, but not for training time as this was considered an opportunity of value to them. This was just above the London Living Wage.

### 2.6 Researcher training

The clinician held a training day for researchers to make changes and improve the information materials and the interview schedule, as well as to provide interview training. This included a practical session when people practised using the digital voice recorders and interviewed each other. There were a role play and discussion on how to help interviewees feel relaxed and talk freely, without going into traumatic events. The researchers read and signed the consent forms themselves at the session, before recording began, and this was useful in learning how to administer the forms to other participants. Seven researchers attended the training, but the following day one of them left a message to say she did not want to participate. The remaining six people took part in data collection and analysis. Four were women, and two were men. In terms of ethnic origin, one was white, three black, one Latin American and one Middle Eastern origin. Three had did not have English as a first language.

### 2.7 Volunteer training for researchers

A second training was delivered by the volunteer co‐ordinator and a SLaM volunteer in the regular format used by them for all volunteer training. The topics covered were as follows:

- An introduction to volunteering within the Trust.
- Communication and listening.
- Mental health issues.
- Boundaries and confidentiality.
- Safeguarding.

Case studies were used to enable people to think about real situations which might arise to make their learning more meaningful. This session was crucial in equipping the patient researchers to carry out the interviews in an ethical and effective manner.

### 2.8 Researcher changes to methods

In the first training session, there was evidence that the new researchers were thinking about how to negotiate their new role. Rather than being patients with a clinician having the decision‐making power, some of the researchers wanted to change this dynamic. During a discussion on the questions to be asked of participants, one researcher asked: ‘How much can we control what happens and how much do you direct it?’ They made a major change to the methodology in that the clinician had intended them to interview other patients outside the researcher group, but they insisted that they would also interview each other first: ‘What about us – don't we get a chance to have our say? What about interviewing each other; would that be useful?’ This was beneficial to the research in that they were able to run through the questions and familiarize themselves with the audio recorder equipment with each other, before going on to interview participants they had not met before. It also gave them a chance to experience being interviewed.

When the interviews were done there was often a long discussion between the researcher and the interviewee before the audio recorder was switched on. While some of this was necessary for information and signing the consent form, the discussion may have included aspects the former patients did not want to be heard. The analysis of the interviews is reported in another paper.^22^

### 2.9 Group meetings with researchers

The training session with researchers and three post‐interview feedback meetings with them were all recorded and transcribed. A subsequent analysis meeting with all six researchers was recorded and transcribed during which they were asked ‘How did you feel about doing the research?’ The meetings totalled about 6 hours and 44 minutes.

They had a research function similar to that of focus groups,^29^ in providing complex data from a range of participants, while empowering them to speak out about their shared experiences. They also had a clinical aim in that they were intended to ensure patient safety and well‐being, both for participants for and researchers.

## 3 ANALYSIS

The aim of this study section was to explore whether a participatory methodology contributed to the promotion of recovery for researchers with a previous diagnosis of complex post‐traumatic stress disorder, CPTSD. A data‐driven approach^30^ was taken to thematic analysis of the transcripts of all meetings with service user researchers. Any mentions of the theme ‘Experiences of the research’ were coded manually by the psychotherapist researcher on NVivo. Extracts were chosen according to the themes they chose, including not only the exact words, but words with similar meanings.

There were 47 extracts in total, and all 6 researchers made more than one contribution. From this overarching theme, 3 subthemes were identified: anxiety about doing the research, identification with the interviewees and an increase in feelings of self‐worth and confidence as a result of doing the research.

### 3.1 Anxiety about doing the research

All the researchers felt anxious about doing the interviews to some degree. Researcher 2 said:

I was really dreading, I was really nervous about coming and doing it and I was rehearsing and rehearsing. I rehearsed to the point of, like, I’m sounding robotic almost, I’m sitting there like it’s just automatic, like I’m reading some sort of script, I’m in a play or something.

Some of the anxiety was about doing something new, as Researcher 1 said: ‘I’m always afraid when I don't know the situation well’. There were also concerns about keeping boundaries in the interviews so that patients did not start to tell their story, but rather were focused on how they felt about their psychotherapy or other treatment. Researcher 1 described her sense of near‐panic: ‘At first I was very nervous and scared and I remember the last day, I was all over the place, wasn't I? And I thought, Jesus, I can't do this today. You know, I couldn't not do it’. There was also a desire to help the clinician: ‘I wanted to do a good job, a good enough job for you and not embarrass myself’.

### 3.2 Identification with interviewees

All the researchers talked about how they had identified with their interviewees and felt empathy for them. Researcher 7, who was allocated an interviewee from his own country, said: ‘I can say it's make me remember because someone coming from the same country as me…I realise the thing I was facing myself, there are many people who are facing the same’. This decreased their sense of social isolation.

Some researchers struggled with taking on the new role:

In a way, I felt, who the hell am I, asking people these questions, you know, I’m no better than them, you know…I said it several times that I was a service user and so forth and so forth. I didn’t want them to think: ‘Oh, someone’s judging me, asking me these questions.

Two researchers struggled because they empathized so much that they wanted to hug their interviewee. Researcher 6 described her experience:

I found the first one quite difficult…I really did want to give her a hug and I can’t remember if I did or not, I don’t think I did, but there was just a connection and I clicked with what her story was about, and I just felt energised by her because she had done so much, I thought it was really, yeah, amazing.

A number of the researchers felt that they could be seen as a helpful role model for their interviewees. Researcher 2 saw it like this:

When they see people like themselves who are on the other side and they’re giving the interview and they feel they’ve come through it, and they feel like there’s some hope there, that you know, they’ve gone through what they’ve gone through…and now they’re sitting there, taking on the role of, not therapist, but an interviewer, but almost like a therapist…’

Researcher 1 added to this: ‘It sort of makes me feel a sort of leadership role…in the sense that it's like I’m helping them to…achieve something…Like a facilitator, you're giving them a stage to say something’. This experience of facilitating others to have their views heard resulted in the researchers’ valuing themselves more highly and recovering some of their self‐confidence.

### 3.3 Increased self‐worth and confidence

One of the purposes of asking former service users to carry out the research interviews was to enable them to connect socially with others through the research project, and thereby promote their emotional health. The project did enable them to connect socially with others, and it was a very specific aspect of the connection which they identified as promoting recovery. This aspect was that they felt they had been given responsibility to help others who had been service users, such as they had once been themselves, and were therefore being recognized as having gone some way towards recovery. Researcher 2 explained it:

Being the interviewer, like, puts us in a position of responsibility which probably helps us as people as well because it makes us feel, not completely recovered, but we’ve gone some way towards recovery and, um, maybe subconsciously or in a placebo way, I’m not sure, yeah, it takes us a step further in terms of our recovery to see how far we’ve come.

Researcher 6, who carried out seven interviews, said:

My experience of it, yeah, kind of similar to what [Researcher 2] said about the position of responsibility and even though I thought I wasn’t as far ahead as I thought I was, sitting in the room and being there, showing up, doing it, kind of showed me: actually, yeah, we can do stuff instead of staying in our own little bubble, so yeah, it was quite nice.

Two researchers said that they had recovered skills that they once had. Researcher 1 said: ‘I think I saw a part of myself that I used to know many years ago’. Researcher 2 was more specific:

…I thought I was utter rubbish at everything…But when I was doing those interviews, because I used to interview people on a daily basis, several of them a day, for years and years over a different issue… I actually enjoyed it. Because I thought: I’m rusty, fair enough, but it kind of gave me confidence back, that you know, there is something I can do.

### 3.4 Clinician observations drawn from reflective log

The psychotherapist kept a reflective log, and in the interests of transparency, some of her observations are included here.

#### 3.4.1 Researcher changes to analysis

‘I (First author) struggled during the research process with my desire to continue with the professional agenda as planned. I had drawn up a list of questions for the semi‐structured interviews to which the researchers made some changes. The questions included the issues which are salient for many clinicians running a trauma service with complex patients:

- Is it always useful to confront the trauma?
- Are stabilization techniques useful on their own or as a treatment adjunct?
- Are groups better than individual therapy for patients?
- What do patients feel about eye movement and desensitization reprocessing, EMDR?
- What do patients feel about narrative exposure therapy?

However, when the patient researchers analysed the transcripts and identified important themes, these were not the themes they identified, and I had to give up the professional clinical role in favour of the researcher collaborator role specified in the methodology’.

#### 3.4.2 Social inclusion

When recruiting participants for the research, the clinician (anon) received an e‐mail which read: ‘The fact that we will all be a part of the system from the inside, will provide a glimpse of how this part of the psychological therapy is run, in regards to the type of trauma you and your team deals with’ (e‐mail 26/10/17). This perception of being ‘a part of the system from the inside’, or socially included, proved crucial during the research process in generating self‐confidence in the researchers. This was the basis for rebuilding other relationships which are part of the healthy attachment necessary for psychological well‐being. One researcher was emphatic about this aspect: ‘If you've got complex trauma, more than one site of the trauma…then what you experience in therapy is not necessarily going to help with all the complexities of it. So if you have another thing outside, like you do volunteering and things like that’.

Researcher 1 was emphatic about wider social connections too, saying: ‘deal with the complex trauma, but don't just do it as a therapy in a room or a one‐to‐one, expand people's opportunity to help themselves a bit more. I feel very, very strongly about that’.

The clinician noted in the reflective log that the researchers began to relate to each other over time, bringing in information which other group members needed, as well as tea and biscuits they thought others would like.

## 4 DISCUSSION

Our findings demonstrate that all six researchers felt that taking part in the research had been a therapeutic process in itself and benefitted their mental health in the ways described above. Once they had overcome their anxiety, the researchers felt that participating in the research signified that they were further on the road to recovery than those they were interviewing because they had been given responsibility. They also recovered lost skills and learned new ones. Given that one of the additional symptoms of the new ICD‐11 diagnosis is ‘persistent beliefs about oneself as diminished, defeated or worthless’,^17^ this is an important finding.

This is in line with the literature on the advantages of participation in research for patients, but has not previously been demonstrated in people with CPTSD difficulties. Springham's research network^15^ included researchers with a range of previous diagnoses who did not investigate their own data. King and Gillard^14^ and their research team did explore personal reflections on the work they did and felt it resulted in the co‐production of knowledge where the researchers were able to move beyond their ‘service user’ identities to bring their skills and expertise to the study. This change was also seen in our study where researchers felt they were modelling recovery or taking on leadership roles.

Our findings show that taking part in research changed the researchers’ feelings about themselves. The group aspect of the research approach where the researcher team met regularly with clinician was effective in enabling the researchers to identify their own agenda rather than adopt that of the clinician, as seen in her reflective log. The researchers then experienced themselves differently, both in relation to the clinician (first author) and the wider world. Kagan et al^5^ describe this process as a ‘new knowledge of the surrounding reality leads to new self‐understanding’.

Our findings also demonstrate that social inclusion through participation was significant for researchers’ mental health. This may be seen through the perspective of social psychologists such as Kagan et al who note how: ‘People who take collective action describe how their sense of belonging and personal worth change for the better’.^5^ This description resonates with the researchers who felt more socially included as a result of their participation in research. It may also be seen from a clinical perspective as the rebuilding of a wider attachment network or social integration. Herman describes this as community re‐integration, the final stage of working therapeutically with complex PTSD.^18^

Managing the clinician's emotional resistance to the patients’ agenda was key to facilitating the emergence of an authentic participation by them. Although it was sometimes a struggle for the clinician to revise her thinking, it was a vital part of genuine collaboration. Frost et al describe this as ‘critical humility’,^7^ a commitment to self‐evaluation and critique.

Academic evaluation of objectivity and potential researcher bias in the health field tends to highlight the disadvantages of a clinician studying patients they have worked with. However, one important advantage may be a pre‐existing relationship of trust. This is affirmed by Liamputtong in her book ‘Researching the Vulnerable’,^31^ where she identifies the establishment of trust as a crucial factor in successful research with vulnerable people. However, it was a limitation of the research that the power imbalance between professionals and researchers was not addressed in terms of structural inequalities, such as class and education, where the professional agenda may be privileged. A future study could also usefully explore the broader role of social and economic factors in the new diagnosis of CPTSD, such as gender relations. The small number of participants was also a limitation as they and their context of inner London may have had specific characteristics which limit generalizability.

## 5 CONCLUSION

A participatory approach to research where patients collaborate with a clinician can contribute to recovery through increasing patient confidence and promoting a social network. This was helpful in the context of the CPTSD symptoms of feeling worthless and interpersonal difficulties. It was particularly important to empower people given their previous experiences of powerlessness and helplessness.

## CONFLICT OF INTEREST

The authors had no conflict of interest in the implementation of the study.
